# Composition Engineering of Indium Zinc Oxide Semiconductors for Damage-Free Back-Channel Wet Etching Metallization of Oxide Thin-Film Transistors

**DOI:** 10.3390/mi14101839

**Published:** 2023-09-27

**Authors:** Xuan Zhang, Sung Woon Cho

**Affiliations:** Department of Advanced Components and Materials Engineering, Sunchon National University, Sunchon 57922, Jeonnam, Republic of Korea; 1215065@s.scnu.ac.kr

**Keywords:** oxide semiconductor, thin-film transistor, compositional engineering, solution process, back-channel wet etching

## Abstract

In contrast to lift-off and shadow mask processes, the back-channel wet etching (BCWE) process is suitable for industrial-scale metallization processes for the large-area and mass production of oxide thin-film transistors (TFTs). However, chemical attacks caused by the corrosive metal etchants used in the BCWE process cause unintended performance degradation of oxide semiconductors, making it difficult to implement oxide TFT circuits through industrial-scale metallization processes. Herein, we propose composition engineering of oxide semiconductors to enhance the chemical durability and electrical stability of oxide semiconductors. The chemical durability of InZnO against Al etchants can be improved by increasing the content of indium oxide, which has a higher chemical resistance than zinc oxide. As a result, A damage-free BCWE-based metallization process was successfully demonstrated for oxide TFTs using In-rich InZnO semiconductors. Furthermore, In-rich InZnO TFTs with wet-etched Al electrodes exhibited electrical performance comparable to that of lift-off Al electrodes, without chemical attack issues.

## 1. Introduction

Multi-component oxide semiconductors (e.g., InGaZnO, InZnSnO, InZnO, and ZnSnO) composed of indium oxide (In_2_O_3_), zinc oxide (ZnO), and tin oxide (SnO) have attracted attention as promising channel materials for high-performance thin-film transistors (TFTs) because of their high electrical mobility and spontaneous n-type semiconducting properties [1]. In contrast to conventional (e.g., Si and Ge) and emerging (e.g., transition-metal dichalcogenide and carbon nanotube) crystalline semiconductors, which have anisotropic conduction pathways, multi-component oxide semiconductors can maintain wide conduction pathways because of the isotropic n^th^ s-orbitals of the metal cations, even in an amorphous state [2,3]. Thus, Amorphous oxide semiconductors can be preferred over other crystalline semiconductors as channel materials for large-area and mass-produced TFT arrays for large-area displays and emerging sensor applications because of their excellent large-area uniformity and low-temperature process compatibility [4,5]. The electrical performance and stability of oxide TFTs have been improved through continuous technological innovations for high-quality oxide semiconductors. They can be fabricated with excellent metal–oxygen–metal (M-O-M) networks, an appropriate electron concentration, and minimal defect states owing to technological developments in the deposition process, post-treatment, channel structure, and material design [6,7].

However, many challenges remain in the metallization of oxide TFTs for the circuit integration of TFT arrays. Lift-off and shadow mask processes that are free of chemical and plasma damage issues can be utilized for the metallization processing of oxide TFTs. In the case of metallization processing using a shadow mask, metal electrodes and metal lines are selectively formed in exposed areas that are not obscured by the shadow mask. Next, in the case of a metallization process using a lift-off process, the patterned photoresists are formed using photolithography, and then a metal film is deposited over the entire sample. Afterward, only the parts with the photoresist pattern are selectively removed through a lift-off process, and metal lines and metal electrodes are selectively formed in the parts without the photoresist pattern. However, they are only suitable for small and laboratory-scale metallization processes. Instead, there are two representative types of industrial metallization processing technologies for circuit integration of oxide TFT arrays: etch stopper layer (ESL) and back-channel wet etching (BCWE) processes [8]. The ESL approach, which uses an insulating etch stopper on the oxide back channel, is suitable for achieving large-area metallization without causing significant damage to the oxide channel. However, mask alignment difficulties and additional parasitic capacitances make it disadvantageous to minimize the TFT size and can lead to difficulties in processing [9,10,11]. The BCWE approach, in which metal electrodes and metal lines are directly defined without a passivation layer, has the advantages of low-cost processing and high-resolution TFT arrays. However, performance degradation of oxide semiconductors after BCWE-based metallization has been observed [12,13]. Because most studies on oxide semiconductor TFTs have utilized laboratory-scale metallization processes to form electrodes, the problems that arise during BCWE-based metallization processes have not been actively studied.

To successfully form an oxide TFT circuit through the BCWE metallization process, high etch selectivity between the oxide semiconductor and the metal electrode/line in the metal etchant is needed. Metal electrodes/lines should be perfectly ionized without the generation of metal residues, while oxide semiconductors should have high chemical durability against metal etchants. Researchers have proposed approaches for improving the etching selectivity between the oxide semiconductor and metal electrode/line. First, to completely ionize a metal electrode without generating metal residues, wet etchants with appropriate pH values must be used, depending on the metal electrode [14,15]. When a metal etchant with an inappropriate pH is used, a large amount of oxide is formed instead of ionizing the metal, which reduces the etching selectivity and significantly affects the electrical properties of the oxide semiconductor. Several methods to improve the chemical durability of oxide semiconductors have been proposed for alleviating chemical damage to the oxide back channel in metal etchants [16,17,18,19,20]. A method of introducing external elements with high chemical resistance (e.g., Sn and Ti) into the interior or surface of an oxide semiconductor was proposed for increasing the chemical resistance [16,17,18]. However, it also makes the channel patterning process for defining the channel area difficult. Additionally, a method was proposed to improve the chemical durability by performing heat treatment for a long period to increase the metal–oxygen (M-O) bonding force and film density [19,20]. However, this method is disadvantageous for the low-cost and mass production of oxide TFT arrays.

In this study, the composition of InZnO semiconductors was controlled for damage-free BCWE metallization of oxide TFTs. In_2_O_3_ and ZnO account for most of the composition of various multi-component oxide semiconductors, such as InGaZnO, InAlZnO, InHfZnO, and InZnO. Therefore, InZnO was selected as the representative oxide material, and the changes in chemical durability with composition control were investigated. According to the Pourbaix diagram, the chemically stable pH regions of binary oxides such as In_2_O_3_ and ZnO can be estimated as follows: In_2_O_3_ (5 < pH < 11) and ZnO (9.5 < pH < 10.5) [21,22]. This indicates that In_2_O_3_ can maintain its original state even in corrosive solutions. Thus, we can expect that the chemical durability of the InZnO semiconductor against metal etchants can be significantly improved by increasing the In content. First, to investigate the pristine electrical properties of the InZnO film, InZnO TFTs were manufactured using a lift-off process without chemical attack problems, and the effects of the composition on the performance were evaluated. Next, the corrosion behavior and chemical durability of the composition-controlled InZnO films were evaluated by monitoring the compositional and microstructural changes before and after immersion in the metal etchant. The chemical durability of InZnO films was improved through compositional engineering and applied to the fabrication of oxide TFTs with wet-etched Al electrodes to realize a damage-free BCWE-based metallization process.

## 2. Experimental Details

### 2.1. Film and Device Fabrication

The precursor solutions for the InZnO films were synthesized by dissolving zinc nitrate hydrate (Zn(NO_3_)_2_·*x*H_2_O; Sigma–Aldrich, St. Louis, MO, USA) and indium nitrate hydrate (In(NO_3_)_3_·*x*H_2_O; Sigma–Aldrich) in 2-methoxyethanol (2ME; CH_3_OCH_2_CH_2_OH; Sigma–Aldrich). InZnO precursor solutions with a molar concentration of 0.1 M were prepared using different composition ratios of In to Zn (In:Zn = 2:8, 4:6, 5:5, 6:4, and 8:2). The synthesized precursor solutions were mixed for 1 h using a sonicator until they were completely and homogeneously ionized. Before spin-coating, they were filtered through a hydrophobic 0.2-μm syringe filter. Individual InZnO precursor solutions were spin-coated at 3000 rpm for 30 s onto heavily doped *p*^++^-Si wafers with 200-nm-thick SiO_2_ layers. Heavily doped *p*^++^-Si wafers and SiO_2_ layers served as the gate electrode and gate dielectric, respectively. The semiconducting InZnO film with superior metal–oxide bonding networks can be produced via sub-sequential soft- (200 °C for 10 min) and hard-bake processes (400 °C for 1 h) using hotplates. The spin-coating and baking processes were each performed twice to form a semiconducting InZnO thin film with a thickness of approximately 10 nm. To confirm the electrical stability of the TFTs, source and drain (S/D) electrodes (100 nm-thick Al) were formed using a conventional lift-off process. The time-dependent transfer characteristics of the InZnO TFTs were evaluated under a positive gate bias stress (PBS, V_GS_ = +10 V) for 7.2 ks. In addition, several InZnO TFTs were fabricated with wet-etched Al electrodes using the BCWE metallization process. For Al metallization through BCWE processing, a 100 nm thick Al film was deposited on the InZnO thin film, and photoresist patterns were partially formed on the Al film. Then, those samples are immersed in a conventional H_2_PO_4_-based acidic Al etchant (pH ≈ 4) for 10 min at 32 °C to define Al S/D electrodes. The electrical performance of InZnO TFTs with wet-etched Al electrodes was measured according to the composition conditions of the InZnO films. The channel dimensions were 500 µm (width) and 50 µm (length).

### 2.2. Film and Device Characterization

The surface morphology and compositional characteristics of the InZnO thin films were evaluated using scanning electron microscopy (SEM; JSM-7610F Plus, JEOL Ltd., Tokyo, Japan) and energy-dispersive X-ray spectroscopy (EDS; JSM-7610F Plus, JEOL Ltd.), respectively. The chemical bonding states of the InZnO films were evaluated via X-ray photoelectron spectroscopy (XPS; ESCALAB 250, Thermo Fisher Scientific, Waltham, MA, USA) using an Al Kα (1486.6 eV) source.

The electrical performance of the InZnO TFTs was evaluated using a semiconductor parameter analyzer (HP-4145B, Agilent Technologies, Santa Clara, CA, USA). The *I_DS_*–*V_GS_* transfer curve was measured with *V_GS_* ranging from −40 to +40 V at a specific *V_DS_* of 10 V. The field-effect mobility (μ*_FE_*) was extracted from the linear regime ((*V_GS_* − *V_Th_*) ≫ *V_DS_*, and *V_DS_* = 1 V) of the transfer curve via the following equation [23]:

(1) $${µ}_{FE}={\left[\frac{L}{C_{i}\;W{\;V}_{DS}}\frac{{dI}_{DS}}{{dV}_{GS}}\right]}_{max},$$

where *C_i_*, *W*, and *L* represent the gate dielectric capacitance per area, channel width, and channel length, respectively.

The threshold voltage (*V_Th_*) was determined from the transfer curve in the saturation regime (*V_DS_* = 10 V) using the following equation [24]:

(2) $$I_{DS}=\left(\frac{W}{2L}\right){µ}_{SAT}\;C_{i}\;{\left(V_{GS}-V_{Th}\right)}^{2},$$

where μ*_SAT_* represents the saturation mobility. *V_Th_* was determined from the x-axis intercept of the √(*I_DS_*) versus *V_GS_* plot via linear extrapolation.

The subthreshold swing (*S*.*S*.) value was extracted from the transfer curve in the saturation regime (*V_DS_* = 10 V) using the following equation [23,24]:

(3) $$S.S.={\left[{\left(\frac{{dI}_{DS}}{{dV}_{GS}}\right)}_{max}\right]}^{-1}.$$

The density of the active subgap defect states (*N_T_*) present at the gate-dielectric/channel interface was extracted from the hysteresis curve using the following equation [25]:

(4) $$N_{T}=C_{i}\;\frac{∆V_{Hys}}{q}.$$

where ∆*V_Hys_* and *q* represent the difference between the *V_Th_* values extracted from the forward and backward transfer curves and the elementary charge, respectively.

## 3. Results and Discussion

**Figure 1:**
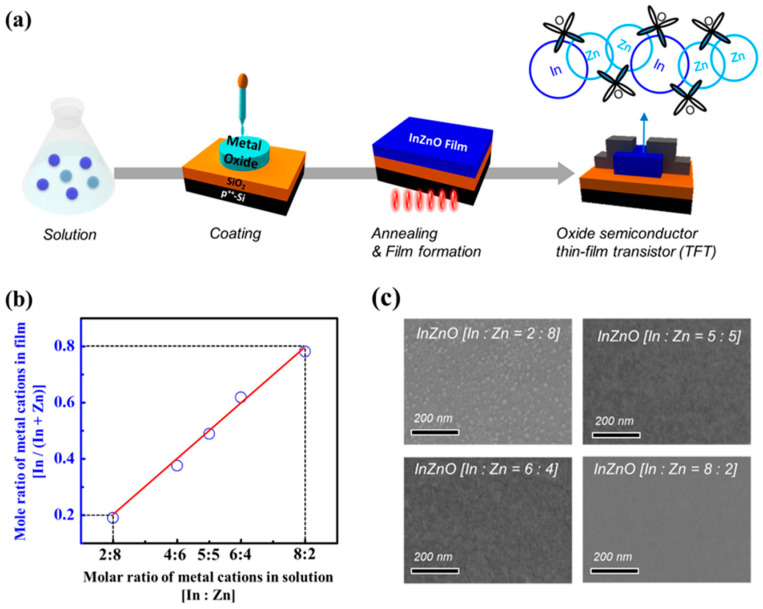
(**a**) Fabrication scheme of solution-processed InZnO semiconductor TFTs; (**b**) molar ratio of metal cations presented in the InZnO film and InZnO solution; (**c**) surface image (top-view) of cation-composition controlled InZnO films.

As shown in Figure 1a, instead of vacuum-based deposition processes, InZnO thin films with various cation compositions were easily fabricated via solution-based coating and annealing. Various precursor solutions with different mixing ratios of In to Zn were synthesized to control the cation compositions of the InZnO thin films. Then, the composition-controlled precursor solutions were uniformly coated via spin-coating and were post-annealed via soft- (200 °C for 10 min) and hard-bake processes (400 °C for 1 h). As shown in Figure 1b and Figure S1, InZnO thin films with composition conditions similar to the molar ratio of the In and Zn precursor solutes added to the precursor solution were prepared. Their compositional characteristics were evaluated using EDS. The InZnO thin films had In:Zn composition ratios of 2:8, 4:6, 5:5, 6:4, and 8:2. The conduction path of the InZnO film, which consists of the outermost orbitals of the In and Zn cations, can be flexibly engineered through solution processing. Regardless of the differences in composition, these films were confirmed to have a thickness of approximately 10 nm using an alpha-step surface profiler. In the SEM images, the In-deficient InZnO film (In:Zn = 2:8) exhibited an island-like crystalline surface morphology (Figure 1c). In contrast, In-equivalent (In:Zn = 5:5) and In-rich (In:Zn = 8:2) InZnO films exhibited smooth surface morphologies characteristic of amorphous films.

**Figure 2:**
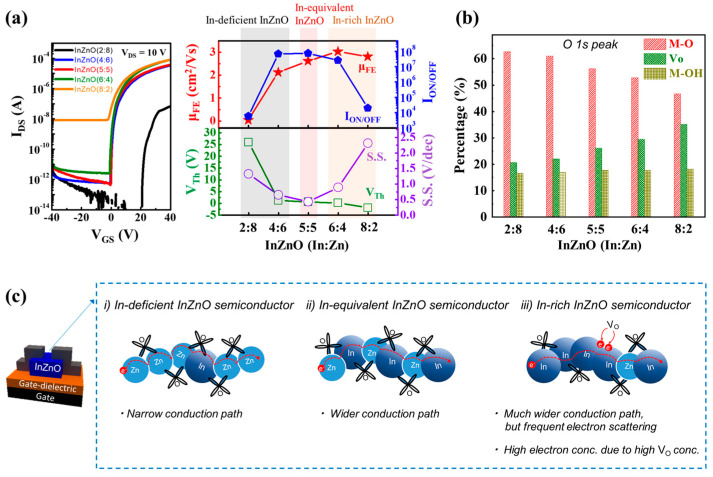
(**a**) Electrical performance of TFTs fabricated using cation-composition controlled InZnO films, including the *I_DS_*–*V_GS_* transfer curve and figures-of-merit (μ*_FE_*, *I_ON/OFF_*, *V_Th_*, and *S*.*S*.); (**b**) chemical bonding states of oxygen elements present in cation-composition controlled InZnO semiconductor films, i.e., M-O bonds, oxygen vacancies (*V_O_*), and O-H bonds; (**c**) electrical conduction path and free electron transport behavior in cation-composition controlled InZnO films.

To explore the pristine electrical properties of the composition-controlled InZnO films, as shown in Figure 2, various InZnO TFTs were fabricated using Al S/D electrodes formed through a lift-off process without chemical damage. The off- and on-current states of all the oxide TFTs fabricated using InZnO films were observed under negative and positive gate bias conditions, respectively (Figure 2a). This indicated that all InZnO thin films have n-type semiconducting properties regardless of the compositional changes. In addition, it was confirmed that all the InZnO thin films were composed of high-quality M-O-M networks with a high content of M-O bonds owing to sufficient thermal annealing, regardless of the composition (Figure 2b and Figure S2). However, the electrical properties of the InZnO TFTs significantly depended on the cation composition. The electrical conduction paths in multi-component oxide semiconductors are formed by the outermost s orbitals of metal cations with different radii. Specifically, the In cations have far wider outermost s-orbitals than the Zn cations. This indicates that the In-rich samples had wider electrical conduction pathways than the In-deficient InZnO samples. As shown in Figure 2c, In-deficient InZnO had narrow conduction pathways because Zn cations with small radii mainly contributed to the formation of the conduction path. Thus, when In-deficient InZnO films were used in the channel region, the TFT performance, such as the on-current level and electrical mobility, was significantly degraded (Figure 2a). In contrast, as shown in Figure 2c, increasing the In content resulted in a wider conduction pathway in the InZnO semiconductor. Therefore, the on-current and electrical mobility of the TFTs were increased by using InZnO films with higher In contents (Figure 2a). When the In ratio was ≥40%, the InZnO TFTs exhibited higher electrical mobility than conventional amorphous Si TFTs. The µ*_FE_* values of InZnO TFTs with In:Zn composition ratios of 4:6, 5:5, 6:4, and 8:2 were 2.1, 2.6, 2.9, and 2.8 cm^2^/V·s, respectively. However, the use of InZnO (In:Zn = 8:2) with an excessive In content caused a high off-current and a negative shift in the threshold voltage. This indicates that excessive oxygen vacancies (*V_O_*) and free electrons exist in In-rich InZnO because the oxidizing power of In (standard oxidation potential of In = +0.34 V) is weaker than that of Zn (standard oxidation potential of Zn = +0.76 V) [26]. Therefore, as shown in Figure 2b, when InZnO (In:Zn = 8:2) with excessive oxygen vacancies and free electrons was used, the electrical performance of the InZnO TFT deteriorated owing to the frequent scattering of excessive free electrons, despite a wide conduction path (Figure 2c).

**Figure 3:**
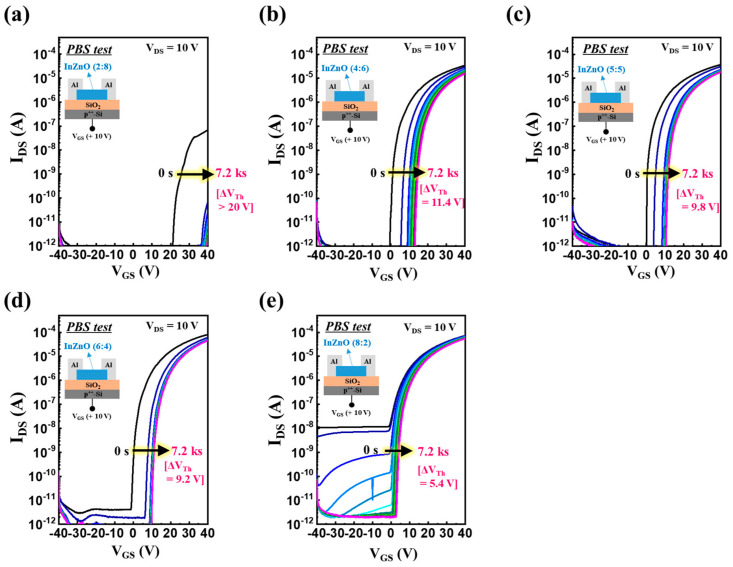
(**a**–**e**) Transfer-curve shifts of oxide TFTs fabricated using cation-composition controlled InZnO semiconductors under PBS tests; the composition ratios of In to Zn (In:Zn) were (**a**) 2:8, (**b**) 4:6, (**c**) 5:5, (**d**) 6:4, and (**e**) 8:2.

Next, as shown in Figure 3, to evaluate the film quality and number of active defects in the composition-controlled InZnO films, the electrical stability of the InZnO TFTs was evaluated via a PBS test. Regardless of the compositional difference between the InZnO films, all the TFTs exhibited a positive shift in the transfer curve. For the In-deficient InZnO TFT, the threshold voltage shifted significantly beyond the measurement range in the positive direction. In contrast, when the In content increased, the electrical stability of the InZnO TFTs was clearly enhanced. When InZnO thin films with In:Zn composition ratios of 4:6, 5:5, 6:4, and 8:2 were used, the changes in the threshold voltage (Δ*V_Th_*) of the InZnO TFT were 11.4, 9.8, 9.2, and 5.4 V, respectively. The positive shift in the transfer curves of the oxide TFTs in the PBS test originated from the charge-trapping phenomenon in the channel region of the oxide semiconductors. Specifically, it arose from two distinct mechanisms: (i) the active defect state of the oxide semiconductor in the channel region and (ii) electron–oxygen gas molecular interactions in the back-channel region [27,28]. The back-channel regions of the InZnO films were exposed to the same ambient conditions without a passivation layer. Therefore, it can be assumed that the difference in electrical stability arose from the active defect states of the oxide semiconductor present in the channel region of the InZnO TFTs. The total number of subgap defect states present in the channel region of InZnO TFTs can be estimated using *S*.*S*. values [23]. The In-rich and In-deficient InZnO films exhibited higher *S*.*S*. values than the other films. This implies that they contained numerous subgap defect states in the channel bulk region and the gate-dielectric/channel interface region.

**Figure 4:**
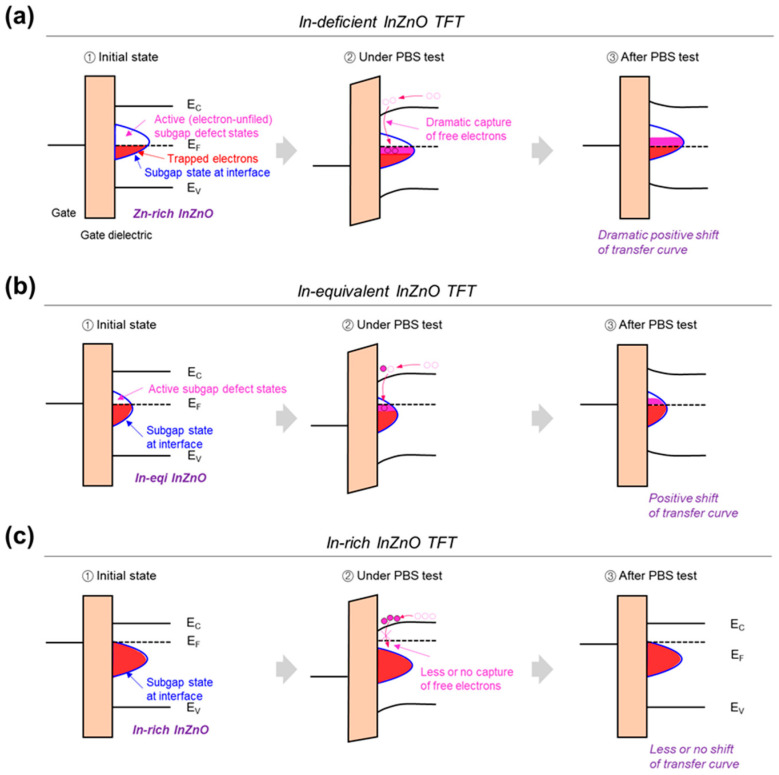
Schematic of the active subgap defect states present at the gate-dielectric/channel interface and the electrical stability of oxide TFTs fabricated using composition-controlled InZnO thin films; (**a**) In-deficient, (**b**) In-equivalent, and (**c**) In-rich InZnO films.

However, as shown in Figure 4, not all the subgap energy states present in the InZnO films can function as charge-trapping states. Only the subgap states that are not filled with electrons can function as active charge-trapping states, causing a significant positive shift in the transfer curve from the PBS test. As shown in Figure 4a, most of the subgap states present in the In-deficient InZnO films exist as electron-unfilled active charge trap states owing to the island-like crystalline film state. Therefore, InZnO TFT with In-deficient InZnO (In:Zn = 2:8) can exhibit severe electrical instability. In contrast, as shown in Figure 4b,c, when the In content increase, the number of electron-unfilled active subgap states decrease, owing to an increase in the free electron density. Additionally, the In-rich InZnO film contains many subgap states owing to the excessive VO concentration; however, they exist as electron-filled inactive charge trap states owing to their high free electron density. Thus, the electrical stability of InZnO TFTs can be improved by introducing In-equivalent and In-rich InZnO semiconductors. Compared with In-deficient InZnO, In-equivalent (In:Zn = 5:5) and In-rich (In:Zn = 6:4 and 8:2) InZnO films are more appropriate for realizing oxide TFTs with good electrical stability. The hysteresis curves confirmed that the density of the active charge-trapping states in the InZnO film was suppressed when the In content increased (Figure S3) [25]. The densities of active subgap defect states in the InZnO films with In:Zn composition ratios of 2:8, 4:6, 5:5, 6:4, and 8:2 were 1.15 × 10^12^, 9.93 × 10^11^, 9.06 × 10^11^, 7.88 × 10^11^, and 3.88 × 10^11^ cm^−2^, respectively.

**Figure 5:**
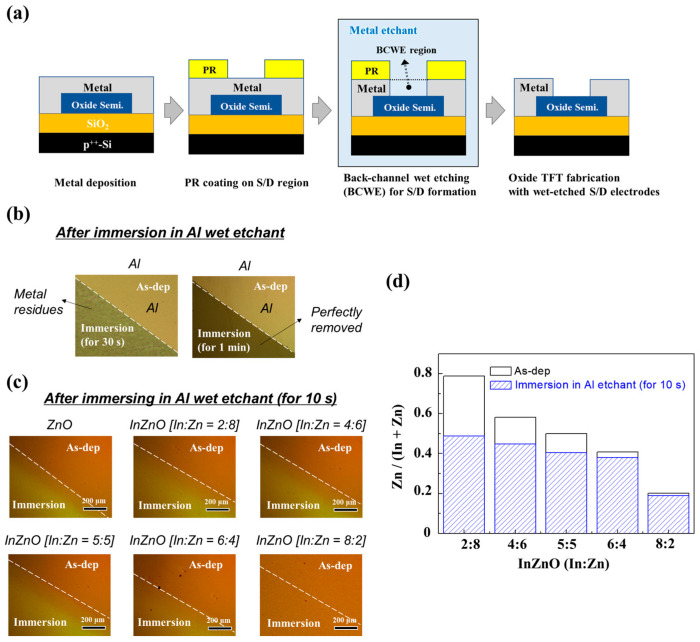
(**a**) Typical sequence of forming S/D metal electrodes of oxide TFTs through the BCWE process; (**b**) OM images taken after immersing Al layers with a film thickness of 100 nm in an Al wet etchant; (**c**) OM images and (**d**) changes in the composition of composition-controlled InZnO films before and after immersion in the Al wet etchant for 10 s.

Figure 5a shows a typical sequence of the BCWE metallization process used to define the S/D electrodes of oxide TFTs. Metals exposed to metal etchants during the BCWE process should be ionized with no metal residues. Typically, for wet etching processing of Al electrodes, H_2_PO_4_-based acidic (pH ≤ 4) or NaOH-based basic (pH ≥ 10) wet-etchants are applied [17,29,30]. In this study, we utilized a conventional H_2_PO_4_-based acidic Al etchant (pH ≈ 4) to define Al S/D electrodes through the BCWE process. The Al layer with a film thickness of 100 nm was completely removed using the H_2_PO_4_-based acidic Al wet etchant within 1 min (Figure 5b). Oxide semiconductors must maintain their original characteristics against chemical attack by metal etchants. If the oxide semiconductor has weak chemical durability against the Al etchant, it will undergo rapid chemical ionization and lose most of its original electrical performance. However, if an oxide semiconductor has sufficient chemical durability, it retains its original electrical and compositional characteristics. Optical microscopy (OM) images confirmed that the In-deficient InZnO and ZnO films were actively corroded by the Al etchants (Figure 5c). Furthermore, it was confirmed that the In-deficient InZnO films underwent rapid compositional changes (Figure 5d and Figure S5). After soaking in the Al etchant, the Zn content ratio was significantly reduced. This indicated that the ZnO present in the In-deficient InZnO films was easily ionized in acidic Al etchants. In contrast, the corrosion behavior of the InZnO films in the Al etchant was significantly suppressed with an increase in the In content. Additionally, it is theoretically well known that In_2_O_3_ can maintain its original chemical state in a wider pH range (5 < pH < 11) than ZnO (9.5 < pH < 10.5) [21,22]. Therefore, it is expected that a damage-free BCWE metallization process can be implemented by applying a chemically durable InZnO film with high In content to the channel area of the TFT.

**Figure 6:**
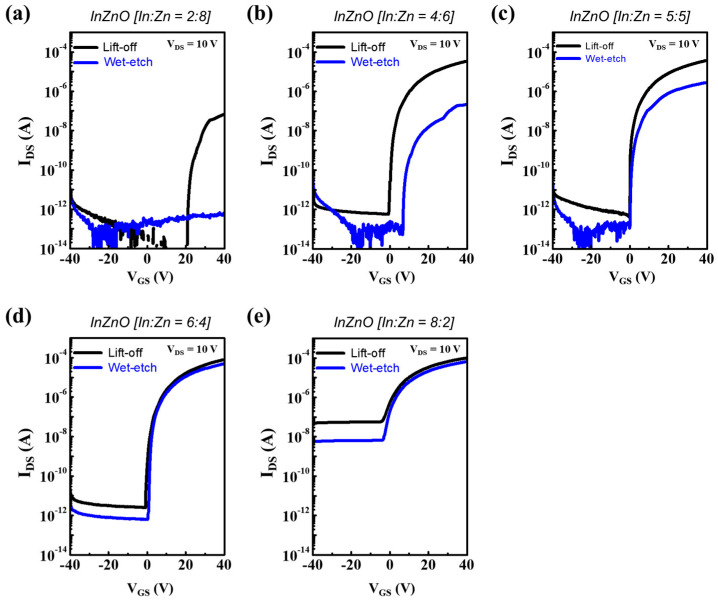
(**a**–**e**) Transfer curves of composition-controlled InZnO TFTs with Al S/D electrodes defined by wet-etching (using an acidic H_2_PO_4_-based Al etchant (pH ≈ 4)) and conventional lift-off processes; the composition ratios of In to Zn (In:Zn) were (**a**) 2:8, (**b**) 4:6, (**c**) 5:5, (**d**) 6:4, and (**e**) 8:2.

Figure 6 and Figure S4 show the electrical performance of various InZnO TFTs with Al S/D electrodes fabricated through wet-etching and lift-off processes. The difference in the electrical properties of InZnO TFTs with different compositions was far larger when a wet-etching process was used than when a lift-off process was used. This indicated that the InZnO films had different chemical durabilities against the same Al etchant depending on their cation compositions. When In-deficient InZnO films were applied, the electrical performance was significantly degraded during BCWE metallization using an acidic Al etchant (Figure 6a,b). However, the electrical deterioration of the TFT during the BCWE metallization process was suppressed as the In content of InZnO increased (Figure 6c–e). This indicates that the chemical durability of InZnO against Al etchants can be optimized through composition control (i.e., composition engineering) and can be significantly improved by increasing the In content. In fact, using In-rich InZnO films (In:Zn = 6:4 and 8:2), excellent electrical performance comparable to that of the original can be achieved in TFTs fabricated with wet-etched Al S/D electrodes. In particular, the wet-etched TFT using InZnO (In:Zn = 6:4) exhibited the best electrical performance (μ*_FE_* = 2.1 cm^2^/V·s and on-current = 50 μA). Thus, a damage-free BCWE-based metallization process using In-rich InZnO semiconductors for oxide TFTs was demonstrated.

## 4. Conclusions

We propose a method for improving the chemical durability of oxide semiconductors for the damage-free BCWE-based metallization of oxide TFTs. In contrast to previously proposed methods of adding chemically durable external elements or providing high thermal energy, we can easily enhance the chemical durability of oxide semiconductors by only manipulating the cation composition of oxide semiconductors. InZnO was selected as a representative oxide material to investigate changes in the chemical durability of oxide semiconductors with composition control. OM images and XPS analysis confirmed that the corrosion behavior of the InZnO film in the Al etchant was significantly suppressed by increasing the content of In_2_O_3_, which has a higher chemical resistance than ZnO. Thus, the chemical durability of the InZnO film against the Al etchant can be significantly improved by increasing the In content. Finally, high-performance oxide TFTs fabricated using In-rich InZnO films with In contents exceeding the Zn contents could be manufactured without degrading the electrical properties through a BCWE-based metallization process. In single-layer oxide semiconductors, there are still limitations in maximizing device performance, electrical stability, and chemical stability simultaneously through composition control. Therefore, in order to overcome the limitations of single-layer oxide semiconductors in the future, it is necessary to develop multi-layer channel structures that stack high-performance oxide semiconductors and chemically durable oxide semiconductors.

## Data Availability

Data are available upon request from the corresponding author.

## Supplementary Materials

The following supporting information can be downloaded at: https://www.mdpi.com/article/10.3390/mi14101839/s1, Figure S1: Composition data and surface images (top view) of cation-composition controlled InZnO semiconductor thin films evaluated by EDS and SEM. The composition ratio of In and Zn (In:Zn) are (a) 2:8, (b) 4:6, (c) 5:5, (d) 6:4, and (e) 8:2. Figure S2: XPS O 1s peaks and deconvoluted fitting curves of cation composition controlled InZnO semiconductor thin films. The composition ratio of In and Zn (In:Zn) are (a) 2:8, (b) 4:6, (c) 5:5, (d) 6:4, and (e) 8:2. Figure S3: Hysteresis properties of InZnO TFTs using cation-composition controlled InZnO semiconductors. The composition ratio of In and Zn (In:Zn) are (a) 2:8, (b) 4:6, (c) 5:5, (d) 6:4, and (e) 8:2. Figure S4: Wet-etching using the H_2_PO_4_-based acidic etchant and lift-off processes of Al source/drain (S/D) electrodes for solution-processed InZnO TFTs. Figure S5: Zn 2p and In 3d XPS peaks obtained from composition-controlled InZnO films before and after immersion in H_2_PO_4_-based acidic Al wet-etchant for 10 s.
