# Supplementary material for: Composition Engineering of Indium Zinc Oxide Semiconductors for Damage-Free Back-Channel Wet Etching Metallization of Oxide Thin-Film Transistors
Source: Micromachines (Basel). 2023 Sep 27;14(10):1839. doi: 10.3390/mi14101839 (PMC10608869; doi:10.3390/mi14101839)
Supplement: Supplementary file 1 [file micromachines-14-01839-s001.zip › micromachines-2633086-supplementary.pdf]

Supporting Information

Composition Engineering of Indium Zinc Oxide Semiconductors for Damage-Free Back-Channel Wet Etching Metallization of Oxide Thin-Film Transistors

Xuan Zhang and Sung Woon Cho *

Department of Advanced Components and Materials Engineering, Sunchon National University, Sunchon 57922, Jeonnam, Republic of Korea; 1215065@s.scnu.ac.kr
* Correspondence: swcho@scnu.ac.kr; Tel.: +82-61-750-5263

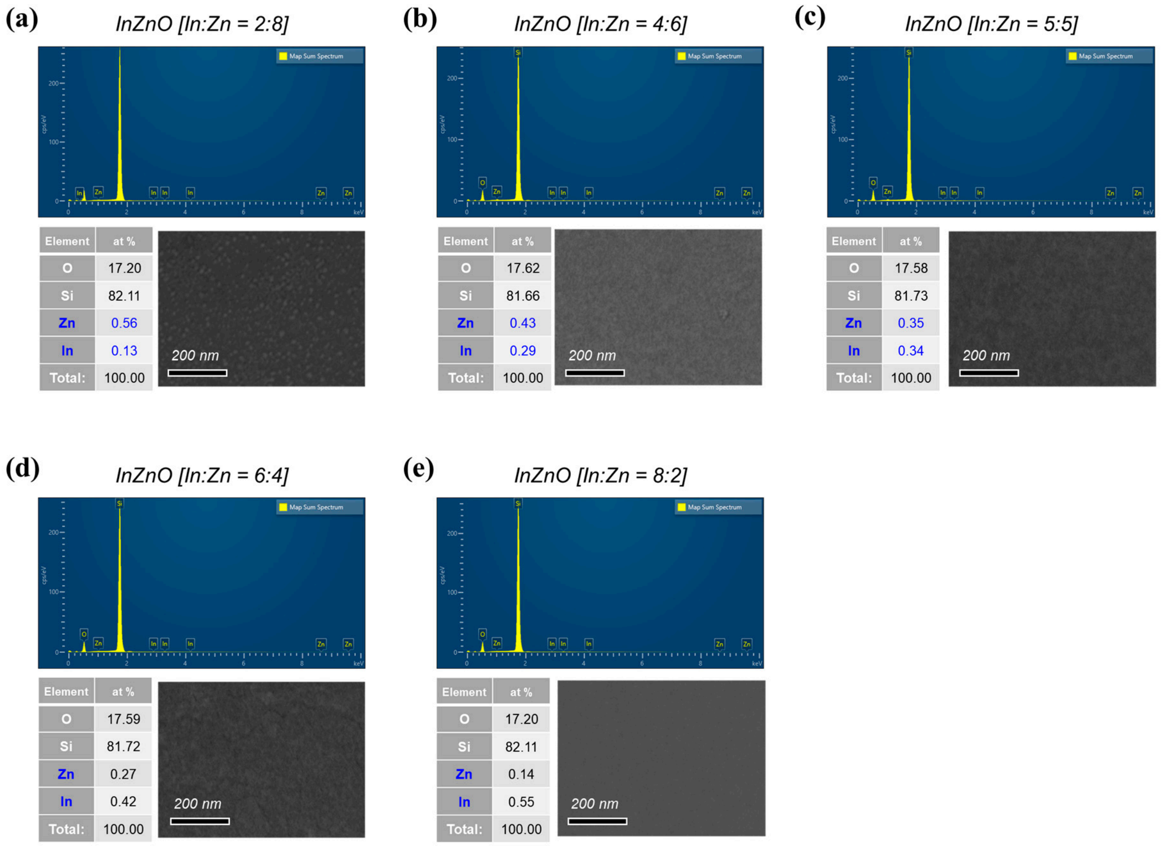

Figure S1. Composition data and surface images (top view) of cation-composition controlled InZnO semiconductor thin films evaluated by EDS and SEM. The composition ratio of In and Zn (In:Zn) are (a) 2:8, (b) 4:6, (c) 5:5, (d) 6:4, and (e) 8:2.

XPS spectra of O 1s peak

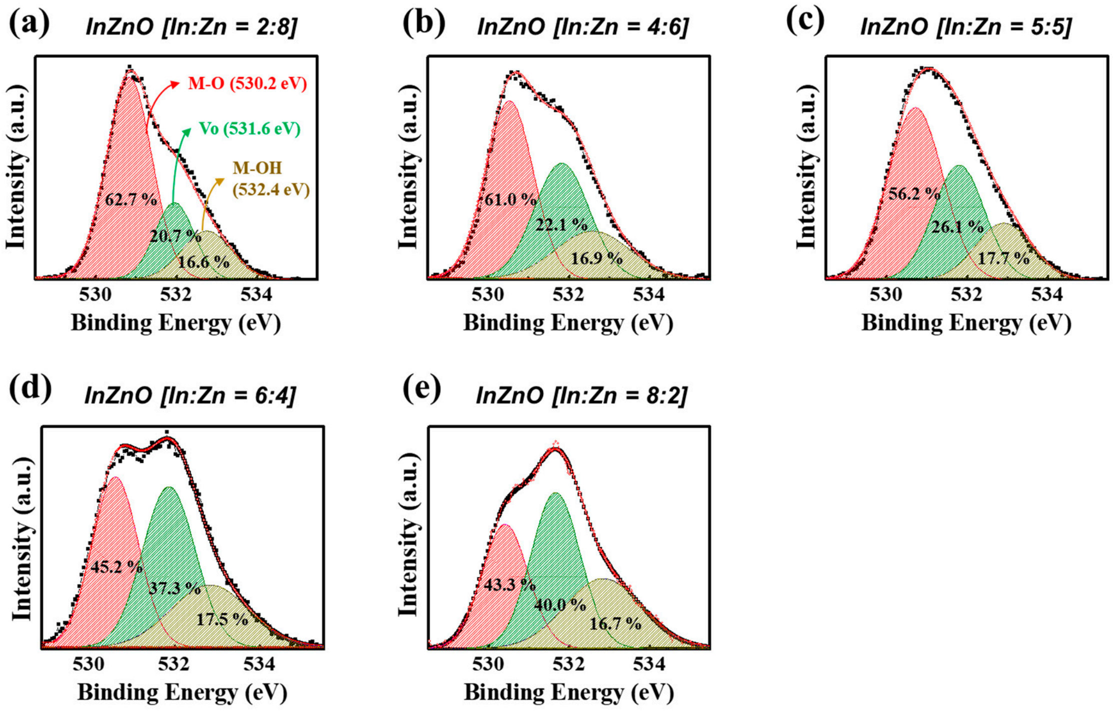

Figure S2. XPS O 1s peaks and deconvoluted fitting curves of cation composition controlled InZnO semiconductor thin films. The composition ratio of In and Zn (In:Zn) are (a) 2:8, (b) 4:6, (c) 5:5, (d) 6:4, and (e) 8:2.

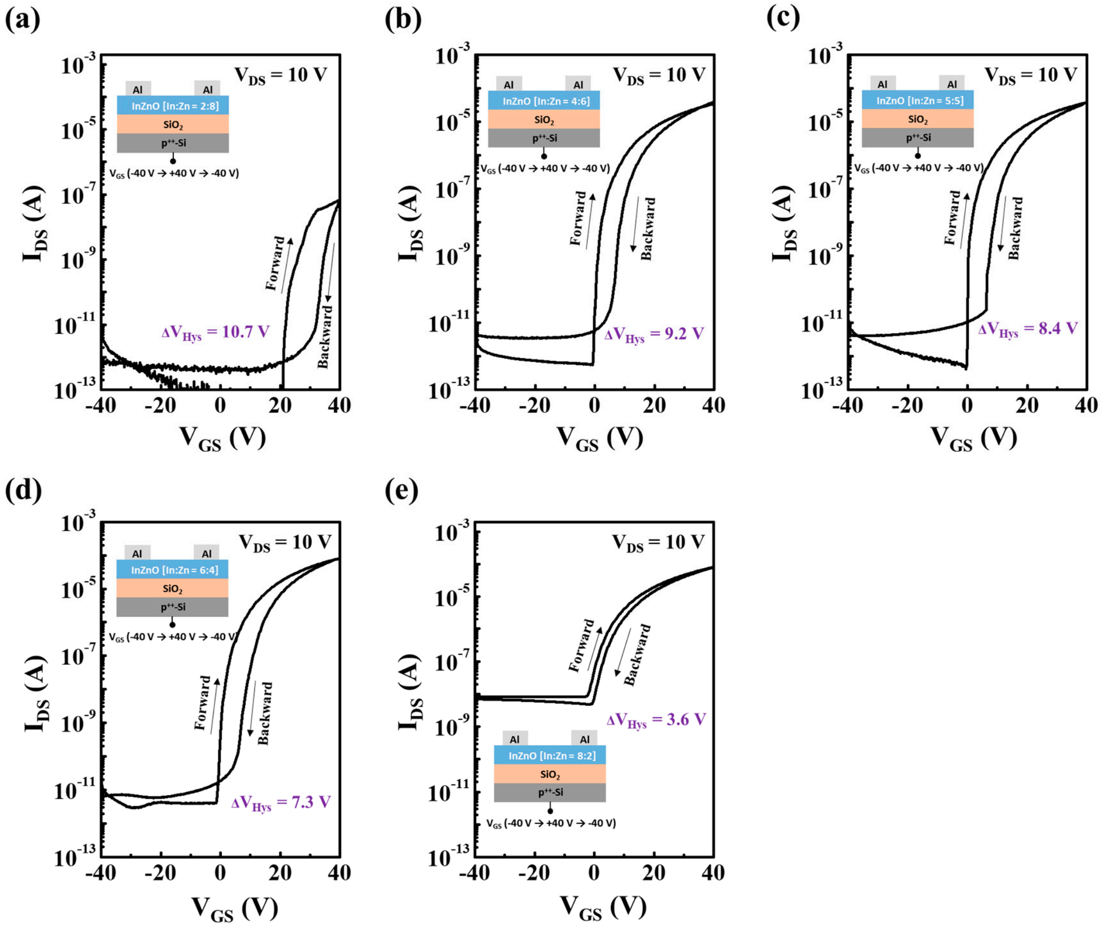

Figure S3. Hysteresis properties of InZnO TFTs using cation-composition controlled InZnO semiconductors. The composition ratio of In and Zn (In:Zn) are (a) 2:8, (b) 4:6, (c) 5:5, (d) 6:4, and (e) 8:2.

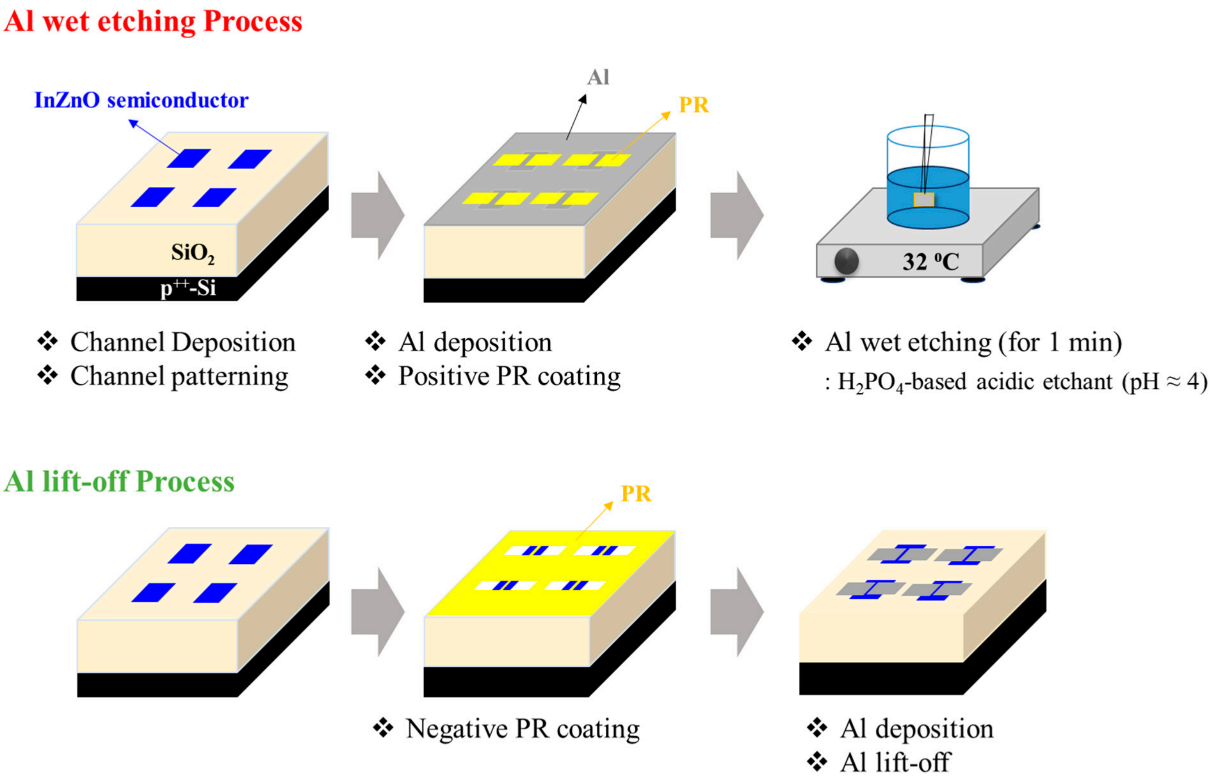

Figure S4. Wet-etching using the H_2PO_4-based acidic etchant and lift-off processes of Al source/drain (S/D) electrodes for solution-processed InZnO TFTs.

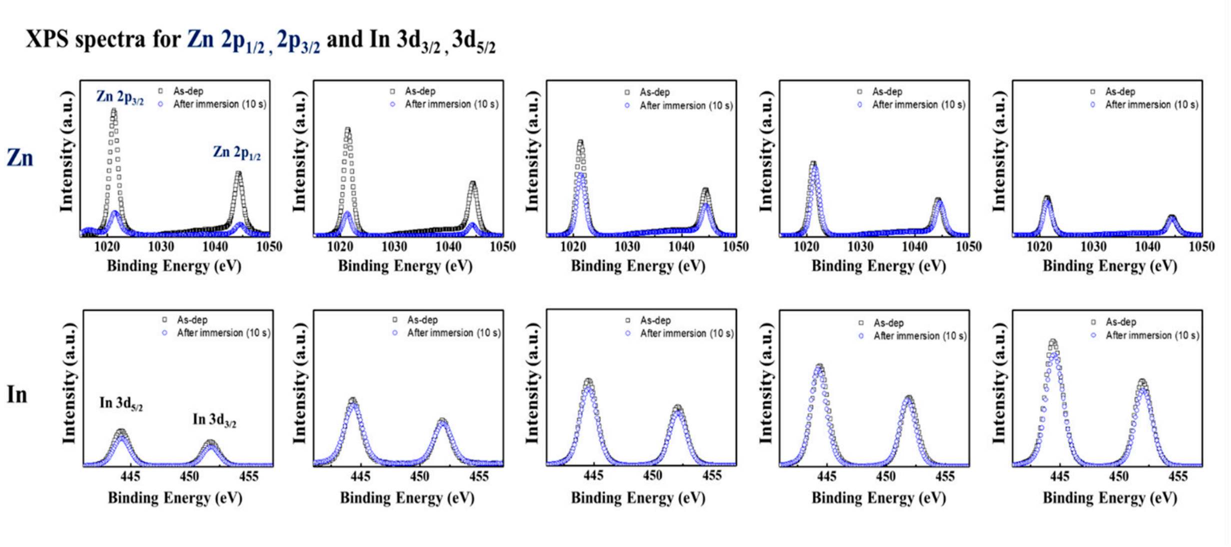

Figure S5. Zn 2*p* and In 3*d* XPS peaks obtained from composition-controlled InZnO films before and after immersion in H_2PO_4-based acidic Al wet-etchant for 10 seconds.
